# Integrating sports pharmacy into pharmacy education: a qualitative study of pharmacy students’ perceptions, barriers, and educational priorities

**DOI:** 10.1007/s11096-025-02086-9

**Published:** 2026-02-24

**Authors:** Kingston Rajiah, Ellen Kirwan

**Affiliations:** School of Pharmacy and Pharmaceutical Sciences, Ulster University, Coleraine, BT52 1SA UK

**Keywords:** Anti-doping, Curriculum development, Pharmacy education, Pharmacy students, Sports pharmacy

## Abstract

**Introduction:**

Sports pharmacy is a developing field in which pharmacists support athlete health, promote safe medication use, and contribute to anti-doping efforts. Despite international recognition of pharmacists’ potential roles, limited evidence exists on pharmacy students’ knowledge and perceptions of this area, particularly in Northern Ireland.

**Aim:**

To explore pharmacy students’ perceptions and experiences of sports pharmacy, with a particular focus on its relevance to pharmacy education, professional roles, and career aspirations.

**Method:**

An exploratory qualitative design was employed, using Braun and Clarke’s inductive thematic analysis. Sixteen MPharm students (Years 1–4) from a university in Northern Ireland participated in semi-structured interviews conducted online between January and May 2025. Data were coded independently by two researchers, with themes developed through consensus.

**Results:**

A total of sixteen students were interviewed. Data saturation was reached at the fourteenth interview, when no new codes or themes were identified, and this was confirmed through researcher consensus following review of coded transcripts. Six key themes were developed from analysis of the full dataset. Interviews lasted between 30 and 45 min (mean length = 37 ± 5 min): (1) Lack of curricular embedding, (2) Latent recognition of professional relevance, (3) Constraints in curriculum integration, (4) Feasible ways for embedding sports pharmacy, (5) Envisioned professional evolution, and (6) Motivation shaped by exposure and identity. Awareness was consistently low across all years. Students recognised sports pharmacy’s value for athlete safety and career development, but expressed concerns about curriculum overload, limited expertise, and variable relevance. Practical solutions included guest lectures, interdisciplinary workshops, and optional or elective modules to provide exposure without adding to the curriculum burden. Sports pharmacy was perceived as an expanding niche with potential to shape future roles for pharmacists.

**Conclusion:**

Pharmacy students valued the potential of sports pharmacy but highlighted challenges in embedding it within an already overloaded curriculum. Flexible and targeted approaches, such as electives and guest lectures, may enhance awareness while accommodating diverse student interests. Future research should evaluate such strategies and their impact on preparing pharmacists for roles in sports pharmacy and anti-doping.

**Supplementary Information:**

The online version contains supplementary material available at 10.1007/s11096-025-02086-9.

## Impact statements

- This research identifies areas where enhanced pharmacist training could reduce the risk of inadvertent doping and medication-related harm in athletes
- Enhanced educational preparation would equip future pharmacists to give safer, evidence-based advice on injuries, rehabilitation, OTC products, and supplement use
- Increased curricular exposure could prepare pharmacists to work effectively within multidisciplinary sports medicine teams, expanding pharmacy’s contribution to athlete health
- Study provides direction for developing competencies and training pathways that support pharmacists in emerging areas of practice, such as anti-doping, supplement stewardship, and sports-related medication management

## Introduction

Pharmacists’ professional roles are expanding in response to evolving healthcare demands, the complexity of medicines, and increasing patient expectations [1]. Within this expansion, sports pharmacy has emerged as a niche but growing field of practice that focuses on the pharmaceutical care needs of athletes and those involved in sport [2]. This includes the safe and effective use of medicines, management of dietary supplements, and the prevention of inadvertent doping. Pharmacists are also well-positioned to provide advice on injury management, rehabilitation, and ethical considerations relating to performance-enhancing substances [3]. Current education and training pathways that support pharmacists in maintaining and enhancing their knowledge and skills, as well as highlighting continuing professional development opportunities within sports pharmacy, are reviewed globally [4].

The World Anti-Doping Code, which standardises anti-doping policies across participating countries and sports, offers pharmacists a chance to recognise sports pharmacy as an emerging speciality and to appreciate the importance of anti-doping education in caring for athlete-patients [5]. This code highlighted the need for healthcare professionals to play a greater role in safeguarding athlete health and ensuring compliance with anti-doping regulations [6]. Pharmacists, as medicine experts, are uniquely positioned to advise on therapeutic use exemptions, assess risks associated with prescription and non-prescription drugs, and provide education on supplements that may contain banned substances [7]. The International Pharmaceutical Federation (FIP) reinforced this position in its statement, outlining pharmacists’ responsibilities in doping prevention, athlete support, and collaboration with anti-doping organisations [8]. However, translating these expectations into practice requires pharmacists to be equipped with adequate knowledge, training, and confidence.

Existing evidence suggests significant gaps in pharmacists’ and pharmacy students’ preparedness for roles in sports pharmacy. A scoping review reported a lack of clear guidelines and insufficient education on pharmacists’ roles in sports pharmacy across multiple countries [9]. In Australia, pharmacists reported lacking the core knowledge and resources required for comprehensive support to deal with doping-related issues [10]. A study from Finland identified pharmacists’ perceptions of insufficient knowledge and identified educational needs that could be integrated into undergraduate and continuing pharmacy education [11]. In Malaysia, community pharmacists stated that they have limited knowledge in the area of doping. Increasing educational programmes and activities on doping and drug use in sports would help enhance pharmacists’ understanding and reduce the risk of inadvertent doping [12]. A study from Qatar showed pharmacy students expressed a strong interest in contributing to doping prevention and promoting the safe and rational use of medicines among athletes. They recommended incorporating dedicated education and training in sports pharmacy within undergraduate pharmacy curricula. [13]. In Japan, pharmacy students stated that they have limited knowledge about doping and are unaware of common violations or hidden risks in medicines and supplements, though many were eager to learn [14]. A study from Norway demonstrated that Pharmacy students have moderate proficiency in sports pharmacy, with only a small proportion having received formal training [15]. Collectively, these studies highlight a global pattern of insufficient training and preparedness in sports pharmacy.

According to FIP, despite the recognition of pharmacists’ potential contributions, sports pharmacy education has not been systematically embedded in pharmacy curricula [16]. Where it does appear, provision is inconsistent and often limited to optional or peripheral teaching [4]. This lack of structured training is problematic given the increasing involvement of pharmacists in multidisciplinary athlete support teams, where they may be responsible for preventing doping violations, advising on supplement safety, and managing therapeutic needs. The absence of a clear educational framework risks undermining pharmacists’ ability to fulfil these responsibilities effectively.

In Northern Ireland, there is limited research exploring pharmacy students’ awareness, perceptions, and educational needs relating to sports pharmacy. Understanding students’ views is critical, as they represent the future workforce, and their perspectives can help inform curriculum development that prepares graduates for evolving roles. Exploring student perspectives also offers an opportunity to identify discrepancies between current training provision and the competencies required to support athletes in both clinical and ethical contexts. This gap is particularly relevant in Northern Ireland, where pharmacists play a central role in primary care and where no published evidence exists on how MPharm students perceive emerging specialist fields such as sports pharmacy. The region has a sporting nature and workforce structure, meaning that understanding students’ preparedness locally is essential for informing curriculum decisions and aligning future graduates with evolving professional expectations.

### Aim

This study aimed to explore pharmacy students’ perceptions and experiences of sports pharmacy, with a particular focus on its relevance to pharmacy education, professional roles, and career aspirations.

## Method

This study was reported in line with the Consolidated Criteria for Reporting Qualitative Research (COREQ) 32-item checklist [17].

### Study design

An exploratory qualitative design was used, guided by Braun and Clarke’s inductive thematic analysis approach. This method was chosen because it allows themes to emerge directly from participants’ accounts rather than applying pre-existing frameworks. Given that pharmacy students’ perspectives on sports pharmacy education are under-researched, this flexible and data-driven approach was well-suited to the study aim. An inductive design was adopted to allow students’ perspectives to emerge without being constrained by pre-existing theoretical constructs. Therefore, behavioural frameworks were not used to guide the development of the interview schedule. These frameworks were instead applied post-hoc in the discussion to help interpret the behavioural dimensions of the findings.

### Research setting

The study was conducted at Ulster University, one of two institutions in Northern Ireland offering the MPharm degree. Ulster University was selected because it provided full access to students across all year groups within the project’s limited timeframe, ensuring feasible recruitment and a comprehensive exploration of perceptions within a single accredited programme.

### Participant recruitment

A purposive sampling approach, informed by the principle of information power, was employed to ensure variation by year of study and gender. Eligible participants were students currently enrolled in the four-year Master of Pharmacy (MPharm) programme who were willing to take part in an individual interview. Students on a leave of absence or not enrolled in the programme were excluded. Students were invited through email and in-class announcements. Those interested contacted the research team through email to express their willingness to participate. From this volunteer pool, participants were purposively selected to ensure representation across all four-year groups and an equal balance of male and female students, consistent with the principles of information power.

### Data collection

Semi-structured interviews were conducted between January and May 2025. Sixteen students participated, with data saturation reached at the 14th interview when no new codes or themes were emerging; this was confirmed by researcher consensus during transcript review. All interviews were carried out online using Microsoft Teams®. An interview guide (Supplementary File 1), informed by existing literature, was developed [5, 13, 14, 18]. It included thirteen open-ended questions. The guide was refined through team discussion and piloted with two students to check clarity, flow, and timing; minor adjustments were made before formal data collection. Although the interview schedule contained 13 open-ended questions, the interview duration (30–45 min) was sufficient several questions elicited brief factual responses (e.g., prior awareness), allowing more time for in-depth exploration of perceptions, barriers, and educational needs. Participants were generally concise and focused on their explanations, enabling comprehensive coverage of all core topics within the allocated time. Interview flow was flexible, and probing questions were used selectively to deepen relevant areas without extending interview burden. Interviews were audio-recorded with consent, and transcribed verbatim. Pilot interviews were excluded from the final analysis. All interviews were conducted by the second author (EK), a final-year Master of Pharmacy (MPharm) student trained in qualitative research methods under the supervision of the first author (KR), an experienced pharmacy education researcher. Peer-to-peer interviewing was considered beneficial in promoting openness and rapport. To minimise potential response bias, participants were reminded that the interviewer had no role in their teaching or assessment, that participation was voluntary, and that their responses would remain confidential. All interviews were audio-recorded with consent and transcribed verbatim.

### Data analysis

Data were analysed using Braun and Clarke’s six-step framework for thematic analysis: familiarisation, coding, searching for themes, reviewing themes, defining/naming themes, and producing the report [19]. NVivo 14 software supported coding and data management. Two researchers coded transcripts independently before comparing and discussing their analyses to reach consensus on codes and themes. Any differences in coding were resolved through discussion, with a third reviewer available for adjudication; however, all discrepancies were successfully resolved through consensus between the two primary coders. An inductive approach was maintained throughout, with themes grounded in participants’ accounts rather than imposed from theoretical models. Themes were developed inductively from the data without reference to pre-existing theoretical frameworks. Behavioural models such as Capability, Opportunity, Motivation – Behaviour (COM-B) and the Theoretical Domains Framework (TDF) were drawn on only at the interpretation stage in the Discussion, to situate the findings and consider implications for curriculum development, rather than to structure coding. For reporting quotations, each participant was assigned a unique identifier (P01–P16) to enhance anonymity and improve readability. Year of study and gender were not retained in the identifiers to reduce deductive disclosure.

### Trustworthiness and rigour

Lincoln and Guba’s four criteria for trustworthiness: credibility, transferability, dependability, and confirmability guided the study [20]. Credibility was enhanced through researcher triangulation during coding and regular team discussions to refine emerging themes. Dependability was supported by maintaining an audit trail documenting analytic decisions. Confirmability was ensured through reflexive discussions, which helped the team remain aware of potential bias. Transferability was strengthened by providing rich detail about the study context and participant demographics, enabling readers to judge applicability to other settings.

### Reflexivity

The research team included a pharmacy educator and researcher with expertise in public health, environmental sustainability, and pharmacy education. Their positionality may have shaped the research process, particularly data collection and interpretation. To address this, reflexive journaling was used to record assumptions, expectations, and analytic reflections throughout the study. Team discussions were held regularly to explore how personal and professional perspectives might influence interpretation, with analytic decisions made collaboratively to minimise individual bias. The interviewer (EK) also had a personal interest in sport; this was acknowledged during reflexive discussions to minimise the risk of assumptions influencing questioning or interpretation. Although the senior researcher (KR) possesses extensive qualitative research expertise, interviews were conducted by the student researcher (EK) to reduce power imbalance and facilitate peer-to-peer openness. This approach is methodologically recognised for enhancing candour when students reflect on their educational experiences, while training and close supervision ensured that interview quality and rigour were maintained.

### Ethics approval

The study was conducted in accordance with the Declaration of Helsinki and approved by the School of Biomedical Sciences Ethics Filter Committee at Ulster University (FCBMS-24-147, date: 21–03–2025).

## Results

**Fig. 1:**
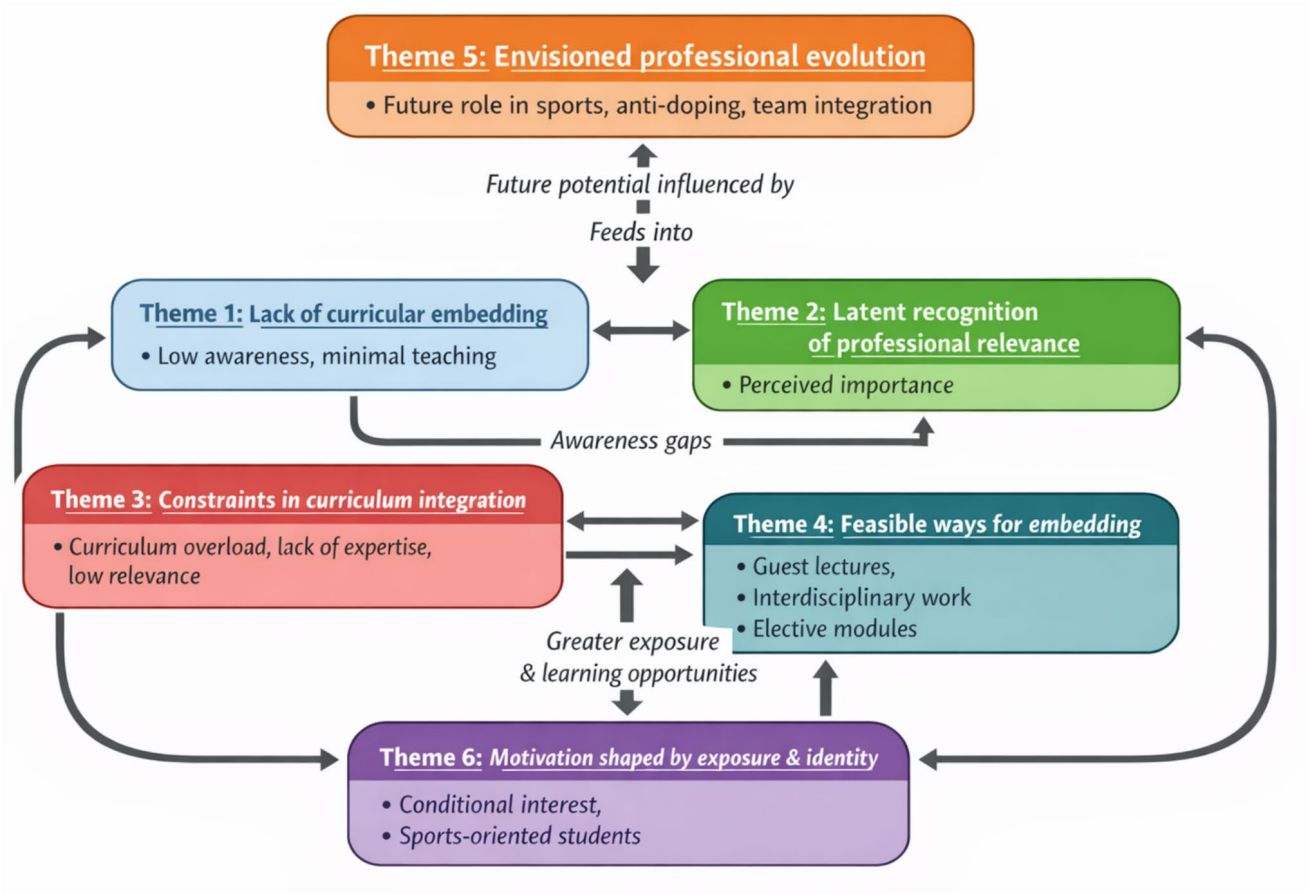
Conceptual model illustrating how pharmacy students understand sports pharmacy. Low curricular embedding (Theme 1) limits awareness, which nonetheless coexists with a latent sense of professional relevance (Theme 2). System-level barriers (Theme 3) restrict integration, whereas practical solutions (Theme 4) offer pathways for embedding sports pharmacy content. Exposure and student identity (Theme 6) mediate motivation and interest, influencing perceptions of future practice (Theme 5). Themes are interconnected, forming a cyclical relationship between awareness, relevance, feasibility, and career motivation

The sample comprised 16 participants, with an equal distribution of male and female students (50% each), and evenly represented across all four year groups (25% per year). Data saturation was reached at the fourteenth interview, when no new codes or themes were identified, and this was confirmed through researcher consensus following review of coded transcripts. Six key themes were developed from analysis of the full dataset. Interviews lasted between 30 and 45 min (mean length = 37 ± 5 min): (1) Lack of curricular embedding, (2) Latent recognition of professional relevance, (3) Constraints in curriculum integration, (4) opportunities for curriculum development, (5) Envisioned professional evolution, and (6) Motivation shaped by exposure and identity. The conceptual map of themes and their interdependencies is provided in Fig. 1.

### Theme 1: Lack of curricular embedding

A strong and consistent finding across all years was students’ limited awareness of sports pharmacy.

#### Sub-theme 1.1: Unfamiliarity with sports pharmacy

Many participants indicated they had never encountered the term before the interview. This was particularly evident among first-year students:

*“I’m not sure, to be honest. I’ve never really heard of sports pharmacy”* (P01)

Similar statements were echoed later in the programme:

*“Not aware at all. I like sport, I play a lot of sport and never heard of it”* (P02)

*“I’m not aware at all and I don’t think many other students are either”* (P03)

Even final-year students described only a very recent introduction:

*“Not very aware because as a fourth-year student, we’ve only received our first lecture on it this year”* (P04)

#### Sub-theme 1.2: Limited information in the curriculum

Students emphasised that sports pharmacy had little to no formal presence within the MPharm programme, noting that teaching was minimal and often incidental. One final-year student described:

*“We’ve only had one lecture in four years on sports pharmacy.”* (P05)

This perception was echoed widely:

*“I don’t think our lecturers would know much about it either… there's not really anything on it in the course.”* (P01)

*“There's so much already in the curriculum that it just never comes up at all.”* (P06)

Together, these accounts demonstrate a consistent belief across all years that opportunities to learn about sports pharmacy are extremely limited.

### Theme 2: Latent recognition of professional relevance

Despite limited knowledge, students across all years acknowledged the importance of sports pharmacy for both professional development and athlete care.

#### Sub-theme 2.1: Protecting athlete safety

Across the dataset, students recognised pharmacists’ potential role in preventing harm, particularly inadvertent doping and unsafe supplement use. One student highlighted:

*“It could help prevent unintentional doping or even just promote athlete safety.”* (P07)

Additional participants expressed similar concerns:

*“Sometimes you wouldn’t be aware of the risks of supplements… pharmacists could help with that.”* (P08)

*“Athletes get drug tested; pharmacists could advise on what you can or can’t take.”* (P16)

These reflections show that even with limited knowledge, students intuitively link sports pharmacy with safeguarding athlete health.

#### Sub-theme 2.2: Expanding career horizons

Students saw sports pharmacy as a way to broaden career pathways and diversify pharmacy roles. As one student noted:

*“It broadens your horizons… it might give people a different perspective on how pharmacy can be studied.”* (P09)

Similarly, others described how the field could open new opportunities:

*“It's definitely different from hospital or community; it offers more career options.”* (P10)

*“If I knew more about it, I think I’d like to explore it because I like sports and pharmacy.”* (P11)

These accounts illustrate a clear belief that sports pharmacy could enhance career diversity, particularly for students with a personal interest in sport.

#### Sub-theme 2.3: Relevance to sports-oriented students

Participants personally involved in sport expressed particular interest:

*“Many pharmacy students would probably be athletes themselves… education keeps competitions fair”* (P13)

*“Sports pharmacy could help athletes with injuries, supplements and treatments”* (P12)

Across all years, students viewed sports pharmacy as a potentially valuable component of education, linking it to athlete well-being and professional diversity.

### Theme 3: Constraints in curriculum integration

Students highlighted several obstacles that limit the inclusion of sports pharmacy in pharmacy programmes.

#### Sub-theme 3.1: Curriculum overload

The most frequent concern was the already saturated curriculum:

*“The curriculum is so big… people may think it’s not important”* (P02)

*“Since the curriculum is so big as it is… people may think it's not important, but there’s just a lot more things we need to cover.”* (P06)

This reinforces the perceived heaviness of the curriculum and student concerns about prioritisation.

#### Sub-theme 3.2: Lack of expertise and resources

Students noted a lack of specialist staff and resources:

*“There aren’t any lecturers or staff members who specialise in sports pharmacy”* (P05)

*“Finding lecturers and finding people that are able to teach it… the resources just aren’t really there.”* (P14)

These further support the concern about lack of specialist staff.

#### Sub-theme 3.3: Low relevance

Some doubted whether all students would engage:

*“The main barrier is that students wouldn’t be willing to engage, thinking it irrelevant”* (P15)

First-year students were more tentative, highlighting relevance as a challenge:

*“I suppose getting people to know of its relevance is the key”* (P16)

These reflections suggest both systemic and perceptual barriers to curriculum integration.

### Theme 4: Feasible ways for embedding sports pharmacy

Despite the barriers, students across years suggested creative approaches to incorporating sports pharmacy.

#### Sub-theme 4.1: Guest lectures and workshops

Students consistently expressed that short, interactive sessions would be more engaging than standalone lectures:

*“Even if it was just workshops or activities… something that gets everyone involved would help.”* (P08)

*“Getting speakers in or even doing field-based activities would make it more real for us.”* (P09)

These preferences reflect a desire for applied practice-oriented learning.

#### Sub-theme 4.2: Interdisciplinary collaboration

Students also highlighted interprofessional learning as a meaningful way to understand sports-related care:

*“If you did it with physio or nutrition students, like one of those multidisciplinary classes, that would make sense.”* (P01)

*“If it was integrated with physios, we’d learn from them and they’d learn from us… that would be very useful.”* (P11)

This reinforces the view that sports pharmacy naturally aligns with collaborative care models.

#### Sub-theme 4.3: Optional/elective modules

Final-year students proposed more formal options:

*“Integration into existing modules using sports-related case studies”* (P05)

*“Even just bringing it into one of our modules… making it part of the professional practice classes would make it more visible.”* (P10)

This strengthens the sub-theme by showing students proposing integration into specific modules.

### Theme 5: Envisioned professional evolution

Students envisaged sports pharmacy as an expanding area of practice with relevance to athlete care and anti-doping.

#### Sub-theme 5.1: Expanding niche

Students perceived a growing need:

*“It’s probably just going to become broader because medicines and sports popularity is always evolving”* (P12)

*“I feel like the uptake could get bigger if people were more educated on it”* (P02)

#### Sub-theme 5.2: Integration with sports teams

The idea of pharmacists as part of multidisciplinary sports teams was frequently raised:

*“A sports pharmacist could be part of the team like physios or dietitians”* (P09)

*“Sports pharmacy could be integrated into sports teams, injury management and supplement use”* (P11)

#### Sub-theme 5.3: Anti-doping and ethical responsibilities

Some highlighted pharmacists’ responsibility for clean sport:

*“Like the Olympics, athletes get drug tested… pharmacists could advise what you can or can’t take”* (P07)

*“I remember getting a talk once about what you can and can’t take before a match… it made me wonder why cold and flu tablets weren’t allowed. Pharmacists should know these things.”* (P16)

This reinforces students’ recognition of pharmacists’ roles in anti-doping guidance.

### Theme 6: Motivation shaped by exposure and identity

Students’ interest in sports pharmacy as a career was mixed, but many acknowledged its potential to influence career choices if education were improved.

#### Sub-theme 6.1: Conditional interest

Students repeatedly explained their interest depended on receiving more exposure during training:

*“If I was more exposed to it, I might choose it instead of hospital or community.”* (P13)

*“If I knew what it involved, I’d probably look into it more… I’d need to know something about it first.”* (P02)

These comments strengthen the argument that motivation is shaped by awareness and experience.

#### Sub-theme 6.2: Sports-oriented students

Students personally engaged in sport showed greater enthusiasm:

*“If I was educated more, maybe I would be inclined to go down that route”* (P01)

*“I really like sports, so I’d probably look into it now to see if there is any future in it for me.”* (P14)

This strengthens the link between personal sporting identity and interest in sports pharmacy.

#### Sub-theme 6.3: Influence of awareness on career choices

Exposure was considered a driver of career pathways:

*“If I was exposed more, I might choose it instead of hospital or community”* (P08)

*“Exposure would increase the interest in pursuing sports pharmacy as a career”* (P15)

These accounts suggest that education and awareness are critical in shaping future career interest in sports pharmacy.

## Discussion

Students’ low awareness reflects a structural absence of sports pharmacy within the curriculum, supporting the argument that capability and opportunity constraints outlined in the COM-B model are key determinants of engagement. Students reported structural barriers, such as curriculum overload and lack of specialist expertise, alongside educational opportunities including guest lectures, interdisciplinary teaching, and elective modules. Many also envisioned pharmacists playing a growing role in sports teams and anti-doping initiatives. Interest in sports pharmacy careers appeared conditional on exposure, indicating that curricular innovation could cultivate future specialists in this emerging field.

Although participants’ limited awareness of sports pharmacy may initially appear unsurprising, this finding carries broader implications that extend beyond the local context. Internationally, pharmacists are increasingly recognised as playing key roles in exercise-related health, anti-doping education, supplement safety, musculoskeletal self-care, and athlete counselling. These responsibilities are embedded within global frameworks such as the World Anti-Doping Code and are not well integrated into pharmacy practice in several countries [9–15]. Despite the expansion of responsibilities, most MPharm programmes, both locally and internationally, provide little structured teaching in sports pharmacy. Therefore, students’ unawareness is not merely a knowledge gap but reflects a wider misalignment between emerging professional expectations and current pharmacy curricula.

Understanding students’ perceptions is particularly important because pharmacy graduates form the future workforce expected to meet these evolving responsibilities. Without adequate exposure during training, graduates may lack the competence to advise on supplement safety, identify doping risks, or support community-based physical activity initiatives. Inadvertent doping remains a recognised public health concern affecting both amateur and elite athletes, and pharmacists are well-positioned to mitigate these risks, provided they are appropriately trained [5–8]. The findings, therefore, highlight the educational need to integrate sports-related competencies into pharmacy programmes to ensure alignment with contemporary professional demands.

Sports pharmacy also intersects with broader educational priorities, including public health promotion, interprofessional practice, and medication-related harm reduction [1–4]. The lack of awareness observed in this study suggests that students may be missing opportunities to develop transferable competencies relevant to these areas. The relevance of this study thus lies in identifying an overlooked domain of pharmacy practice and highlighting the need for curricular innovation not only within one institution but across pharmacy education more widely.

The findings clearly show that pharmacy students across all years possessed minimal understanding of sports pharmacy, with many encountering the term for the first time during the interview. Even final-year students described only limited or incidental exposure. This suggests that sports pharmacy remains largely neglected within current curricula. Expected progressive growth in knowledge across the degree was not evident, as students in later years expressed uncertainty comparable to earlier-year students. These observations echo previous studies reporting knowledge deficits among both pharmacy students and practising pharmacists [13, 14]. The findings reinforce global evidence that sports pharmacy is insufficiently embedded within pharmacy education. Without structured learning opportunities, students are unlikely to develop either a foundational or advanced understanding of the field. This is concerning given pharmacists’ growing involvement in supporting athletes, preventing inadvertent doping, and advising on safe use of medicines and supplements [4]. Failure to incorporate sports pharmacy into curricula may therefore leave future pharmacists underprepared for responsibilities increasingly recognised by professional bodies and international guidelines.

From an educational perspective, these findings strengthen the case for curriculum reform. Introducing sports pharmacy early in pharmacy programmes, with reinforcement across subsequent years, would support progressive development of knowledge. Even basic teaching on definitions, relevance to athlete health, and the role of pharmacists within sport could help bridge current gaps. Without intentional inclusion, graduates may continue to lack awareness of a field gaining international prominence.

Although the study was not theoretically driven, interpreting the findings through COM-B and TDF highlights practical levers for curriculum development. Behaviour appears shaped by capability, opportunity, and motivation: capability was limited by minimal curricular representation; opportunity was constrained by curriculum overload and a lack of specialist educators; and motivation was conditional, arising mainly among students with personal interest in sport. The Theoretical Domains Framework (TDF) further points to deficits in knowledge, skills, social/professional role identity, and environmental context as barriers to engagement. Applying these behavioural perspectives helps explain the persistently low awareness and clarifies mechanisms that must be addressed to facilitate the uptake of sports pharmacy education.

Despite their low baseline awareness, students acknowledged the potential importance of sports pharmacy, particularly for safeguarding athlete health and broadening professional opportunities. A key theme was pharmacists’ role in preventing inadvertent doping caused by inappropriate medication or supplement use. This aligns with international literature emphasising pharmacists’ responsibility in supporting clean sport. The FIP guidelines [17] highlight pharmacists’ duty to differentiate legitimate from prohibited substances and educate athletes on safe medication practices. A global review similarly positions pharmacists as essential members of Athlete Support Personnel (ASP), helping ensure compliance with anti-doping regulations while promoting athlete health [4]. Students’ recognition of these roles, despite minimal prior exposure, indicates an intuitive understanding of pharmacists’ potential contribution.

Students also noted that sports pharmacy could offer alternative career pathways beyond traditional community and hospital sectors. This aligns with studies reporting that pharmacists are interested in sports-related roles but feel underprepared due to inadequate training [9]. Students with personal involvement in sport expressed greater motivation and found sports pharmacy particularly meaningful, echoing studies showing that sporting identity increases interest in anti-doping education [14]. These insights suggest that embedding sports pharmacy content could resonate strongly with specific student groups while raising general awareness among others.

Students identified several barriers to integrating sports pharmacy into the curriculum, including curriculum overload, limited expertise, and low perceived relevance. Curriculum overload was the most dominant concern, consistent with broader debates in pharmacy education where expanding professional roles compete for limited teaching time [21]. Given the crowded nature of the MPharm curriculum, adding new standalone modules is unlikely to be feasible. Instead, sports pharmacy could be incorporated within existing teaching areas by using sports-related case studies may offer a pragmatic solution. For example: Medicines optimisation and therapeutics can include cases involving athletes, injury management, and return-to-play considerations; Public health and health promotion modules can incorporate physical activity guidance, supplement use, and doping-risk education; Law, ethics, and governance teaching can integrate anti-doping regulations and responsibilities of healthcare professionals; Interprofessional education sessions can include joint teaching with physiotherapy, sports science, and nutrition students. This integrated approach supports exposure without requiring additional curricular space.

The lack of specialist expertise was another barrier, with students doubting whether the faculty possessed sufficient knowledge to teach the subject. Addressing this may require guest lecturers, partnerships with sports organisations, or CPD-trained academic staff. Low perceived relevance also emerged, with students suggesting that interest may depend on personal involvement in sport. This echoes findings that student motivation towards doping education varies with sporting engagement [14]. This constraint, however, does not preclude feasible approaches. Guest lecturers from sports medicine, physiotherapy, anti-doping agencies, or sports nutrition can provide relevant input, even where specialist sports pharmacists are few. National and international bodies, including the FIP, offer an additional platform for developing foundational training materials, competency frameworks, and introductory modules that institutions can adopt or adapt. Collaborations with organisations such as national anti-doping agencies, sports science departments, and ASP networks may also help bridge expertise gaps. In this way, the absence of local specialists need not limit students’ exposure, as educational provision can be supported through cross-disciplinary and international partnership models.

Despite recognising barriers, students offered pragmatic suggestions for integrating sports pharmacy. Guest lectures and workshops were the most frequently proposed approaches, offering targeted exposure without adding substantial curriculum burden. Short interventions have been shown to improve awareness in emerging areas of pharmacy practice, particularly when specialist expertise is limited [22]. Students also advocated interdisciplinary education with physiotherapy, nutrition, or sports science students, aligning with global calls for interprofessional education (IPE) to enhance collaborative practice [23]. Elective modules were proposed to offer deeper learning for interested students without increasing pressure on all learners. Such electives have been shown to expand the scope of pharmacy practice and open new career pathways [24].

Students across all years saw strong potential for sports pharmacy to develop as a professional niche, identifying roles in athlete support teams, anti-doping initiatives, and broader sports medicine services. Their views mirror global trends, highlighting pharmacists’ growing contributions to sports medicine and clean sport. Students described pharmacists working with physiotherapists, dietitians, and other ASP members, reflecting existing multidisciplinary models in elite sport where pharmacists’ expertise in safe medication and supplement use is highly relevant [9, 11].

Interest in sports pharmacy careers was often conditional, hinging on exposure and prior awareness. Students with sporting backgrounds demonstrated stronger motivation, while others expressed interest only after learning about potential roles during the interview. This pattern mirrors literature showing that a lack of exposure limits awareness and interest in anti-doping education [14]. These findings suggest that curricular exposure could shape career interests and help develop future specialists in sports pharmacy. Without formal integration into pharmacy education, this emerging field risks remaining overlooked.

### Limitations of the study

This study has several limitations. It was conducted within a single university in Northern Ireland, which may limit the transferability of findings to other pharmacy programmes in the UK or internationally. The views expressed may not capture the full diversity of student perspectives. There is a possibility of self-selection bias, with students more open to discussing new areas of pharmacy practice being more likely to take part. The study relied on self-reported accounts, which are subject to recall bias and social desirability bias. Although the interview guide was informed by previous literature, it did not undergo formal content validation through expert review by sports pharmacists or anti-doping specialists. Cognitive interviewing with target participants was also limited, although two students piloted the guide for flow and clarity. As such, some relevant areas may not have been fully explored, and the phrasing of certain questions may have influenced the depth or direction of responses.

Despite these limitations, the study provides valuable insights into an underexplored area of pharmacy education. Despite being situated within a single institution, several findings are transferable to other pharmacy programmes internationally. Many of the structural issues identified, such as crowded curricula, limited specialist expertise, and low baseline awareness of emerging practice areas, are widely reported across pharmacy education literature. The lack of formal sports pharmacy teaching observed in this study aligns with international evidence indicating that most pharmacy programmes do not explicitly address anti-doping, supplement safety, or athletes’ medication needs. Similarly, students’ conditional motivation, reliance on personal sporting identity, and interest driven by exposure reflect behavioural mechanisms (capability, opportunity, motivation) that are broadly applicable across contexts. These shared features suggest that the challenges and opportunities identified in this study are not unique to Northern Ireland but reflect systemic gaps in preparing future pharmacists for evolving roles in sports medicine and athlete support. As such, the insights generated have relevance for global discussions on curriculum development, professional training standards, and the integration of emerging fields within pharmacy education.

### Implications for policy and practice

These findings have clear implications for pharmacy education and professional policy. Applying the COM-B model and TDF highlights practical levers for curriculum development. Capability can be strengthened through structured teaching on anti-doping, supplement safety, and sports-related case studies. Opportunity may be improved by collaborations with sports science, physiotherapy, and anti-doping organisations, helping to address current gaps in expertise. Motivation can be enhanced through role modelling, guest speakers, and elective pathways that make sports pharmacy more visible and engaging. These behaviourally informed strategies offer feasible, scalable approaches for embedding sports pharmacy without adding substantial curricular burden. The consistently low awareness among students demonstrates the need to integrate sports pharmacy more explicitly into pharmacy curricula. Embedding sports-related examples within existing modules may increase relevance while avoiding curriculum overload. Professional and regulatory bodies such as the GPhC and FIP could facilitate this by developing competency frameworks that acknowledge sports pharmacy as part of pharmacists’ evolving scope of practice. For practitioners, the findings highlight the importance of ongoing CPD and collaboration with anti-doping organisations to reduce inadvertent doping and enhance athlete safety.

### Recommendations for future research

Interviews were selected as the initial methodological approach because sports pharmacy is an emerging and under-researched area, and no prior evidence was available to guide the development of structured survey items. Qualitative methods were therefore necessary to explore underlying perceptions, motivations, and contextual factors in depth. The themes generated from this exploratory work can now inform the design of future quantitative surveys aimed at assessing the prevalence of these views across larger student populations. Future research should examine pharmacy students’ perceptions across multiple institutions and countries to identify broader trends. Quantitative surveys could measure awareness, interest, and training needs at scale, while longitudinal studies could explore how exposure influences interest over time. Further research should also include educators and practising pharmacists to assess alignment between student expectations, curricular design, and professional practice in sports pharmacy.

## Conclusion

This study examined pharmacy students’ awareness, perceptions, and educational needs in relation to sports pharmacy. Although awareness was low across all year groups, students recognised its potential value in protecting athlete health, preventing inadvertent doping, and creating new career opportunities. At the same time, they raised concerns about curriculum overload, limited expertise, and whether the subject would be relevant for everyone. Sports pharmacy was seen as an emerging role within the profession, but students felt its integration into education should be flexible and targeted. Students’ suggestions included optional or elective modules, guest lectures, and interdisciplinary workshops, which would allow interested students to explore the area without adding pressure to an already full curriculum. Adopting such approaches could increase awareness of this growing field while respecting the diverse interests of pharmacy students.

## Supplementary Information

Below is the link to the electronic supplementary material.

Supplementary file1 (DOCX 29 KB)

## Data Availability

The datasets generated during and/or analysed during the current study are available from the corresponding author on reasonable request.

## References

[1] Muscat NA, Sinclair P, Zapata T, et al. Embracing pharmacists’ roles in health-care delivery. Lancet Reg Health Eur. 2024;46:101088. 10.1016/j.lanepe.2024.101088. 39529814 10.1016/j.lanepe.2024.101088 PMC11551493

[2] Bomfim JHG. Pharmaceutical care in sports. Pharmacy. 2020;8:218. 10.3390/pharmacy8040218. 33207610 10.3390/pharmacy8040218 PMC7712766

[3] Hooper AD, Cooper JM, Schneider J, et al. Current and potential roles in sports pharmacy: a systematic review. Pharmacy. 2019;7:29. 10.3390/pharmacy7010029. 30875783 10.3390/pharmacy7010029 PMC6473300

[4] Anderson A, May C, Stuart M, et al. Sports pharmacy practice and education: a global overview. International Pharmaceutical Federation; 2022. https://www.fip.org/. Accessed 14 Nov 2025.

[5] Kawaguchi-Suzuki M, Anderson A, Suzuki S. Reconsidering sports pharmacists and anti-doping education as the world celebrates the Olympic and Paralympic Games. Am J Pharm Educ. 2021. 10.5688/ajpe8695. 34544745 10.5688/ajpe8695 PMC8499666

[6] World Anti-Doping Agency. The World Anti-Doping Code. 2021. https://www.wada-ama.org/en/what-we-do/world-anti-doping-code. Accessed 14 Nov 2025.

[7] Hooper AD, Marquez J, Bajorek B, et al. Enhancing pharmacists’ engagement and collaboration in sport and exercise medicine: an intervention mapping study using the behaviour change wheel. Explor Res Clin Soc Pharm. 2025;19:100619. 10.1016/j.rcsop.2025.100619. 40548337 10.1016/j.rcsop.2025.100619 PMC12178790

[8] International Pharmaceutical Federation. The role of the pharmacist in the fight against doping in sport. 2014. https://www.fip.org/file/1513. Accessed 14 Nov 2025.

[9] Greenbaum DH, McLachlan AJ, Roubin RH, et al. Pharmacists supporting athletes: a scoping review exploring the knowledge, role and responsibilities of pharmacists in preventing the unintentional use of prohibited substances by athletes. Int J Pharm Pract. 2022;30:108–15. 10.1093/ijpp/riac010. 35262700 10.1093/ijpp/riac010

[10] Greenbaum DH, McLachlan AJ, Roubin RH, et al. Examining pharmacists’ anti-doping knowledge and skills in assisting athletes to avoid unintentional use of prohibited substances. Int J Pharm Pract. 2023;31:290–7. 10.1093/ijpp/riad015. 36869840 10.1093/ijpp/riad015

[11] Lemettilä M, Leppä E, Pohjanoksa-Mäntylä M, et al. Anti-doping knowledge and educational needs of Finnish pharmacists. Perform Enhanc Health. 2021;9:100195. 10.1016/j.peh.2021.100195.

[12] Voravuth N, Chua EW, Mahmood TMT, et al. Engaging community pharmacists to eliminate inadvertent doping in sports: a study of their knowledge on doping. PLoS ONE. 2022;17:e0268878. 10.1371/journal.pone.0268878. 35687540 10.1371/journal.pone.0268878 PMC9187095

[13] Awaisu A, Mottram D, Rahhal A, et al. Knowledge and perceptions of pharmacy students in Qatar on anti-doping in sports and on sports pharmacy in undergraduate curricula. Am J Pharm Educ. 2015. 10.5688/ajpe798119. 26689844 10.5688/ajpe798119 PMC4678744

[14] Shibata K, Ichikawa K, Kurata N. Knowledge of pharmacy students about doping, and the need for doping education: a questionnaire survey. BMC Res Notes. 2017;10:396. 10.1186/s13104-017-2713-7. 28800770 10.1186/s13104-017-2713-7 PMC5554002

[15] Dabrowska N, Malmberg L, Nejati H, et al. Competence in sports pharmacy among pharmacy students in Norway. Pharmacy. 2024;12:3. 10.3390/pharmacy12010003. 10.3390/pharmacy12010003 PMC10801607 38251397

[16] International Pharmaceutical Federation. Sports pharmacy practice and education. 2022. https://farmaciavirtuale.it/wp-content/uploads/2022/12/3606-Sports-pharmacy-practice-and-education-Fip.pdf. Accessed 14 Nov 2025.

[17] Tong A, Sainsbury P, Craig J. Consolidated criteria for reporting qualitative research (COREQ): a 32-item checklist for interviews and focus groups. Int J Qual Health Care. 2007;19(6):349–57. 10.1093/intqhc/mzm042. 17872937 10.1093/intqhc/mzm042

[18] Nicholls AR, Lazuras L, Petrou M, et al. A systematic review on the effectiveness of anti-doping education for university students. Emerging Trends Drugs Addict Health. 2025;5:100168. 10.1016/j.etdah.2024.100168.

[19] Braun V, Clarke V. Using thematic analysis in psychology. Qual Res Psychol. 2006;3:77–101. 10.1191/1478088706qp063oa.

[20] Lincoln YS, Guba EG. Naturalistic inquiry. Beverly Hills: SAGE; 1985.

[21] Ozucelik Y, Collins JC, Pace J. Identifying the educational needs of pharmacists engaging in professional development: a global systematic review. J Am Pharm Assoc. 2025;65:102418. 10.1016/j.japh.2025.102418. 10.1016/j.japh.2025.102418 40349977

[22] Forsetlund L, O’Brien MA, Forsén L, et al. Continuing education meetings and workshops: effects on professional practice and healthcare outcomes. Cochrane Database Syst Rev. 2021. 10.1002/14651858.CD003030.pub3/full. Accessed 15 Nov 2025. 10.1002/14651858.CD003030.pub3 PMC8441047 34523128

[23] Spaulding EM, Marvel FA, Jacob E, et al. Interprofessional education and collaboration among healthcare students and professionals: a systematic review and call for action. J Interprof Care. 2021;35:612–21. 10.1080/13561820.2019.1697214. 31865823 10.1080/13561820.2019.1697214 PMC7305974

[24] Awaisu A, Khalifa S, Mottram D, et al. Instructional design and assessment of an elective course on the use of drugs in sport. Curr Pharm Teach Learn. 2018;10:1124–31. 10.1016/j.cptl.2018.05.020. 30314549 10.1016/j.cptl.2018.05.020

