# Supplementary material for: Integrating sports pharmacy into pharmacy education: a qualitative study of pharmacy students’ perceptions, barriers, and educational priorities
Source: Int J Clin Pharm. 2026 Feb 24;48(3):909–20. doi: 10.1007/s11096-025-02086-9 (PMC13176078; doi:10.1007/s11096-025-02086-9)
Supplement: Supplementary file 1 — Supplementary file1 (DOCX 29 KB) [file 11096_2025_2086_MOESM1_ESM.docx]

**Interview Questions (In-depth interview)**

1. From your perspective, what does sports pharmacy practice entail, and how do you see it fitting into the broader field of pharmacy?
2. Why do you think sports pharmacy education is important for pharmacy students?
3. How aware do you feel pharmacy students are about the field of sports pharmacy practice and the opportunities it offers?
4. Have you personally been involved in any activities or initiatives related to sports pharmacy during your pharmacy education?
5. In your opinion, what are some of the main challenges or barriers to integrating sports pharmacy practice and education into pharmacy curricula?
6. Have you encountered any specific challenges in trying to learn more about sports pharmacy during your studies?
7. What opportunities do you see for pharmacy education programs to better incorporate sports pharmacy principles and skills into their curricula?
8. Can you suggest any innovative approaches or solutions that could help promote sports pharmacy education among pharmacy students?
9. Looking ahead, how do you envision the role of pharmacists specializing in sports pharmacy evolving in the future?
10. What changes would you like to see in pharmacy education and practice to further emphasize sports pharmacy?
11. Are you personally interested in pursuing a career in sports pharmacy, and if so, what aspects of it appeal to you the most?
12. How do you think exposure to sports pharmacy education during your pharmacy studies might influence your future career choices?
13. Is there anything else you would like to add or discuss regarding pharmacy students' views on sports pharmacy practice and education?
